# A real‐world study of glucocorticoid treatment in COVID‐19 patients with different disease severities

**DOI:** 10.1002/ctm2.235

**Published:** 2020-12-08

**Authors:** Xiaofei Jiao, Ya Wang, Dan Liu, Shaoqing Zeng, Jianhua Chi, Ruyuan Li, Yang Yu, Ruidi Yu, Siyuan Wang, Yuan Yuan, Yue Gao, Sen Xu, Chunrui Li, Qinglei Gao

**Affiliations:** ^1^ National Medical Center for Major Public Health Events Tongji Hospital, Tongji Medical College Huazhong University of Science and Technology Wuhan People's Republic of China; ^2^ Department of Hematology Tongji Hospital, Tongji Medical College Huazhong University of Science and Technology Wuhan People's Republic of China

Dear Editor,

The dramatic rise in confirmed coronavirus disease 2019 (COVID‐19) cases poses a rigorous challenge to the global healthcare system. Previous studies have indicated that the cytokine storm plays a major role in the progression and death of patients diagnosed with COVID‐19.^1^, ^2^ Therefore, glucocorticoids as an immunomodulatory therapy may be beneficial.^3^, ^4^ However, evidence concerning glucocorticoids for patients with COVID‐19 is controversial and limited by small sample sizes or flawed study designs.^5^, ^6^, ^7^, ^8^, ^9^ A recent randomized controlled trial (RCT) showed that 6 mg of dexamethasone once per day for ten days reduced deaths by one‐third in ventilated COVID‐19 patients.^10^ However, the practical application of glucocorticoids in clinical treatment has not been clearly stated. Considering the gap between RCT participants and actual clinical users, we believe it is of great value to explore the application of glucocorticoids and their effectiveness on patient prognosis in the real world based on elaborated information from electronic medical records.

Herein, we implemented a real‐world, multicenter study with comprehensively detailed clinical data of 2044 patients with COVID‐19 who had been discharged or died from January 27 to March 21, 2020 in the Sino‐French New City campus and the Optical Valley Campus of Tongji Hospital in Wuhan, China. All patients were classified into the noncritical group or critical group based on their most severe condition during the entire course of disease (Supporting Information Methods). The flowchart is shown in Figure S1. We aimed to depict the administration of glucocorticoids in a large population. We employed multivariate logistic regression and Cox regression to explore whether glucocorticoids affect the prognosis of patients with COVID‐19.

The detailed demographic and clinical characteristics of patients with different severities are shown in Tables S1 and S2. The use of glucocorticoids was heterogeneous in patients in the two groups. Glucocorticoids were especially widely used in critical patients compared with noncritical patients (83.6% vs 24.9%, *P *< .001). The critical patients received glucocorticoid therapy earlier after illness onset (1.0, IQR [interquartile range]: 1.0‐3.0 vs 2.0, IQR: 1.0‐4.0, *P *= .002), and the treatment duration was shorter (5.0, IQR: 3.0‐10.0 vs 8.0, IQR: 5.0‐12.0, *P *< .001). The recommended days of glucocorticoid use and the timepoint at which to initiate use remain inconclusive.

A further comparison between glucocorticoid users and nonusers is presented in Table 1. In the noncritical patients, the instability of vital signs in users was noticeable, including higher temperature (*P *< .001), faster respiratory rate (*P *< .001), lower mean arterial pressure (*P *= .009), and reduced SpO2 (*P *< .001). More antibiotics and intravenous immunoglobin were received by users than by nonusers (*P *< .001; *P *< .001, respectively). The mortality rates of the users and nonusers were similar (0.7% vs 0.2%, *P *= .168). However, the incidence of various complications of the users was significantly higher. The median hospital length of stay was significantly prolonged by nearly one week in users (24.0, IQR: 19.0‐32.0 vs 18.0, IQR: 12.0‐25.0, *P *< .001), as well as the time from illness onset to discharge or death (36.0, IQR: 29.0‐43.0 vs 35.0, IQR: 27.0‐43.0, *P *= .003). In the critical patients, the mortality rates were 84.8% for users and 88.6% for nonusers. Similar to noncritical patients, more users received intravenous immunoglobin treatment (*P *= .001). This finding suggested that immunomodulatory therapy may be an important method to treat COVID‐19. The users among critical patients also experienced a remarkably prolonged hospital length of stay (12.0, IQR: 6.5‐21.5 vs 5.5, IQR: 4.0‐17.0, *P *= .001), especially for survivors (34.0, IQR: 28.5‐39.5 vs 21.0, IQR: 20.5‐25.5, *P *= .003).

**TABLE 1:** Comparison between glucocorticoid‐users and nonusers in the noncritical group and critical group

	Noncritical (N = 1776)			Critical (N = 268)		
	Users (N = 443)	Nonusers (N = 1333)	*P* value	Users (N = 224)	Nonusers (N = 44)	*P* value
Age, years	61.0 (51.0‐69.0)	61.0 (49.0‐69.0)	.865	69.0 (62.0‐77.0)	69.5 (62.0‐78.5)	.823
Sex	‐	‐	.028	‐	‐	.494
Female	217 (49.0%)	735 (55.1%)	‐	79 (35.3%)	13 (29.5%)	‐
Male	226 (51.0%)	598 (44.9%)	‐	145 (64.7%)	31 (70.5%)	‐
Presence of comorbidity	245 (55.3%)	718/1330 (54.0%)	.660	175/222 (78.8%)	37 (84.1%)	.540
Hypertension	174 (39.3%)	487/1330 (36.6%)	.335	122/222 (55.0%)	27 (61.4%)	.507
Diabetes	82 (18.5%)	199/1330 (15.0%)	.084	46/222 (20.7%)	14 (31.8%)	.117
Coronary heart disease	40 (9.0%)	113/1330 (8.5%)	.769	35/222 (15.8%)	11 (25.0%)	.188
Number of comorbidities	1.0 (0.0‐2.0)	1.0 (0.0‐1.0)	.588	1.0 (1.0‐2.0)	2.0 (1.0‐3.0)	.023
Temperature, °C	38.8 (38.0‐39.0)	38.0 (37.2‐38.8)	<.001	38.4 (37.8‐39.0)	38.3 (37.1‐39.0)	.413
Fever	399/442 (90.3%)	1024/1332 (76.9%)	<.001	189/223 (84.8%)	32 (72.7%)	.078
Cough	317/442 (71.7%)	955/1332 (71.7%)	1.000	182/223 (81.6%)	33 (75.0%)	.404
Dyspnea	213/442 (48.2%)	476/1332 (35.7%)	<.001	152/223 (68.2%)	29 (65.9%)	.860
Respiratory rate, per minute	21 (20‐23)	20.0 (20‐22)	<.001	29 (22‐33)	26 (22‐32)	.538
Mean arterial pressure, mm Hg	95.0 (87.0‐104.0)	96.7 (89.0‐106.0)	.009	97.0 (89.4‐107.6)	97.2 (90.1‐105.5)	.607
SpO_2_, %	95.0 (92.0‐97.0)	96.0 (94.0‐97.0)	<.001	85.0 (76.0‐92.0)	89.0 (78.0‐96.0)	.101
SOFA score at admission	1.0 (0.0‐2.0)	0.0 (0.0‐1.0)	<.001	5.0 (4.0‐6.0)	4.0 (3.0‐8.5)	.968
Time from illness onset to hospital admission, days	10.0 (7.0‐14.0)	13.0 (9.0‐20.0)	<.001	10.0 (7.0‐15.0)	12.0 (7.0‐19.0)	.455
Antibiotics	405 (91.4%)	954 (71.6%)	<.001	218/223 (97.8%)	41 (93.2%)	.128
Antiviral treatments	421 (95.0%)	1251 (93.8%)	.414	187 (83.5%)	33 (75.0%)	.198
Intravenous immunoglobin	173 (39.1%)	147 (11.0%)	<.001	134 (59.8%)	14 (31.8%)	.001
High‐flow nasal cannula oxygen therapy	36/442 (8.1%)	28/1329 (2.1%)	<.001	9 (4.0%)	2 (4.5%)	1.000
Noninvasive mechanical ventilation	0 (0.0%)	0 (0.0%)	‐^†^	96 (42.9%)	21 (47.7%)	.619
Invasive mechanical ventilation	0/442 (0.0%)	1/1329 (0.1%)	1.000	105 (46.9%)	16 (36.4%)	.247
Duration of mechanical ventilation, days	‐	9.0 (9.0‐9.0)	‐^†^	5.0 (2.0‐9.0)	3.0 (1.0‐5.0)	.009
ECMO	0 (0.0%)	0 (0.0%)	‐^†^	6 (2.7%)	1 (2.3%)	1.000
Duration of ECMO, days	‐	‐	‐^†^	12.5 (2.5‐18.0)	1.0 (1.0‐1.0)	.571
The highest SOFA Score	1.0 (0.0‐2.0)	1.0 (0.0‐1.0)	<.001	14.0 (12.0‐17.0)	13.5 (8.5‐16.5)	.310
Death	3 (0.7%)	3 (0.2%)	.168	190 (84.8%)	39 (88.6%)	.643
Acute liver injury	231 (52.1%)	400 (30.0%)	<.001	118 (52.7%)	24 (54.5%)	.870
ARDS	94 (21.2%)	84 (6.3%)	<.001	224 (100.00%)	44 (100.00%)	‐ ^‡^
Respiratory failure	10 (2.3%)	8 (0.6%)	.005	217 (96.9%)	38 (86.4%)	.010
Septic shock	5 (1.1%)	7 (0.5%)	.187	193 (86.2%)	37 (84.1%)	.813
Acute cardiac injury	45/369 (12.2%)	72/1206 (6.0%)	<.001	169/215 (78.6%)	23/41 (56.1%)	.003
Hypoproteinemia	136 (30.7%)	140/1330 (10.5%)	<.001	172 (76.8%)	26 (59.1%)	.017
Admission to ICU	3 (0.7%)	5 (0.4%)	.420	137 (61.2%)	18 (40.9%)	.019
Coagulopathy	31 (7.0%)	56 (4.2%)	.022	136 (60.7%)	20 (45.5%)	.067
Sepsis	181 (40.9%)	262 (19.7%)	<.001	224 (100.00%)	44 (100.00%)	‐ ^‡^
Acute kidney injury	36 (8.1%)	86 (6.5%)	.234	111 (49.6%)	17 (38.6%)	.192
Heart failure	5/442 (1.1%)	8/1332 (0.6%)	.330	106/223 (47.5%)	15 (34.1%)	.135
Secondary infection	2 (0.5%)	5 (0.4%)	1.000	18 (8.0%)	2 (4.5%)	.545
Hospital length of stay, days	24.0 (19.0‐32.0)	18.0 (12.0‐25.0)	<.001	12.0 (6.5‐21.5)	5.5 (4.0‐17.0)	.001
Hospital length of stay of survivors, days	24.0 (19.0‐32.0)	18.0 (12.5‐25.0)	<.001	34.0 (28.5‐39.5)	21.0 (20.5‐25.5)	.003
Hospital length of stay of nonsurvivors, days	9.0 ^§^	9.0 ^§^	.658	10.0 (6.0‐17.0)	5.0 (4.0‐13.0)	.002
Time from illness onset to death or discharge, days	36.0 (29.0‐43.0)	35.0 (27.0‐43.0)	.003	24.0 (18.0‐35.0)	20.5 (13.5‐31.5)	.066
Time from illness onset to discharge, days	36.0 (30.0‐43.0)	35.0 (27.0‐43.0)	.003	48.0 (38.5‐51.0)	41.0 (30.5‐47.0)	.125
Time from illness onset to death, days	22.0 ^§^	15.5 ^§^	.564	22.0 (17.0‐30.0)	19.0 (12.0‐29.0)	.097
Duration of vital shedding after illness onset, days	11.0 (6.0‐17.0)	11.0 (6.0‐17.0)	.727	12.0 (7.0‐19.0)	12.5 (6.0‐20.0)	.983
Time from illness onset to ICU, days	13.0 ^¶^	19.5 (4.0‐27.0)	.857	15.0 (11.0‐21.0)	13.5 (6.5‐23.5)	.398
ICU length of stay, days	13.0 ^¶^	9.0 (1.0‐15.5)	.294	7.0 (3.0‐11.0)	5.0 (4.0‐8.0)	.307

Data are median (IQR), n (%), or n/N (%). *P* values were calculated by the Mann‐Whitney *U* test, χ^2^ test, or Fisher's exact test, as appropriate. Abbreviations: ARDS, acute respiratory distress syndrome; COVID‐19, coronavirus disease 2019; ECMO, extracorporeal membrane oxygenation; ICU, intensive care unit; SOFA, sequential organ failure assessment.

^†^ The Mann‐Whitney *U* test, χ^2^ test, or Fisher's exact test cannot be conducted because no patients received this treatment.

^‡^ The χ^2^ test cannot be conducted because every patient in the critical group had ARDS and sepsis.

^§^ The IQRs of hospital length of stay of nonsurvivors and time from illness onset to death cannot be conducted because only three users and nonusers in the noncritical group died.

^¶^ The IQRs of time from illness onset to ICU and ICU length of stay cannot be conducted because only three users in the noncritical group admitted to ICU.

Some potential factors were found to influence the effectiveness of glucocorticoids in critical patients. The detailed results are displayed in Table 2. A total of 190 of the 224 glucocorticoid users in the critical patients died, while only 34 recovered. The nonsurvivors presented with older age (70.0, IQR: 64.0‐78.0 vs 65.0, IQR: 54.0‐73.0, *P *= .010), lower SpO2 (84.0, IQR: 74.0‐91.0 vs 91.5, IQR: 84.5‐94.0, *P *< .001), and higher SOFA score at admission (5.0, IQR: 4.0‐7.0 vs 4.0, IQR: 3.0‐4.0, *P *= .002). The lymphocyte and platelet counts were both significantly lower in nonsurvivors than in survivors (0.56, IQR: 0.39‐0.80 vs 0.74, IQR: 0.56‐1.06, *P *= .003; 159.0, IQR: 106.3‐224.7 vs 223.5, IQR: 148.5‐316.5, *P *= .002). The level of albumin among nonsurvivors was lower (30.8, IQR: 27.9‐33.6 vs 33.2, IQR: 29.4‐36.8, *P *= .020), and the levels of blood urea nitrogen, creatinine, prothrombin time, D‐dimer, high‐sensitivity cardiac troponin I and NT‐proBNP were all higher in nonsurvivors (*P *< .050). This suggested that abnormal metabolism and coagulation function are related to adverse outcomes of glucocorticoid treatment. The initial levels of C reactive protein, ferritin, procalcitonin, interleukin‐2R, interleukin‐6, interleukin‐8, interleukin‐10, and tumor necrosis factor‐α were remarkably higher in nonsurvivors (*P *< .050), which revealed that the release of excessive inflammatory factors may also influence the effectiveness of glucocorticoids. More research is needed to explore the underlying mechanism and the interaction between cytokines and glucocorticoids. In summary, highly heterogeneous individuals vary in their response to glucocorticoid treatment. Even for patients with the same disease severity, physicians should fully grasp the auxiliary examination results of COVID‐19 patients before the administration of glucocorticoids.

**TABLE 2:** Baseline characteristics of survivors and nonsurvivors in the critical group who received glucocorticoids

	Total (N = 224)	Survivors (N = 34)	Nonsurvivors (N = 190)	*P* value
**Demographic and clinical characteristics**				
Age, years	62.0 (69.0‐77.0)	65.0 (54.0‐73.0)	70.0 (64.0‐78.0)	.010
≥60	181 (80.8%)	22 (64.7%)	159 (83.7%)	.016
Sex	‐	‐	‐	.248
Female	79 (35.3%)	15 (44.1%)	64 (33.7%)	‐
Male	145 (64.7%)	19 (55.9%)	126 (66.3%)	‐
Presence of comorbidity	175/222 (78.8%)	24 (70.6%)	151/188 (80.3%)	.252
Hypertension	122/222 (55.0%)	19 (55.9%)	103/188 (54.8%)	1.000
Diabetes	46/222 (20.7%)	9 (26.5%)	37/188 (19.7%)	.490
Coronary heart disease	35/222 (15.8%)	3 (8.8%)	32/188 (17.0%)	.309
Number of comorbidities	1.0 (1.0‐2.0)	1.5 (0.0‐2.0)	1.0 (1.0‐2.0)	.891
Temperature, °C	38.4 (37.8‐39.0)	38.6 (38.0‐39.0)	38.3 (37.8‐38.9)	.379
Fever	189/223 (84.8%)	28 (82.4%)	161/189 (85.2%)	.795
Cough	182/223 (81.6%)	29 (85.3%)	153/189 (81.0%)	.638
Dyspnea	152/223 (68.2%)	21 (61.8%)	131/189 (69.3%)	.426
Respiratory rate, per min	29 (22‐33)	27 (21‐30)	30 (22‐33)	.185
Mean arterial pressure, mmHg	97.0 (89.4‐107.6)	97.0 (89.8‐106.0)	97.0 (89.3‐107.8)	.999
SpO2, %	85.0 (76.0‐92.0)	91.5 (84.5‐94.0)	84.0 (74.0‐91.0)	<.001
SOFA score at admission	5.0 (4.0‐6.0)	4.0 (3.0‐4.0)	5.0 (4.0‐7.0)	.002
0–1	1 (0.4%)	1 (2.9%)	0 (0.0%)	‐
2–3	52 (23.2%)	14 (41.2%)	38 (20.0%)	‐
≥4	171 (76.3%)	19 (52.9%)	152 (80.0%)	‐
Time from illness onset to hospital admission, days	10.0 (7.0‐15.0)	12.0 (7.8‐16.3)	10.0 (7.0‐14.5)	.354
**Laboratory findings**				
White blood cell count, × 10^9^ per L	9.05 (6.29‐12.75)	8.00 (5.56‐10.51)	9.15 (6.68‐13.09)	.067
Lymphocyte count, × 10^9^ per L	0.59 (0.42‐0.83)	0.74 (0.56‐1.06)	0.56 (0.39‐0.80)	.003
Hemoglobin, g/L	129.0 (116.0‐143.0)	127.0 (114.0‐139.5)	129.0 (116.5‐143.5)	.613
Platelet count, × 10^9^ per L	165.5 (112.8‐234.0)	223.5 (148.5‐316.5)	159.0 (106.3‐224.7)	.002
Alanine aminotransferase, U/L	27.0 (18.0‐41.8)	25.0 (17.5‐44.0)	28.0 (18.0‐41.3)	.894
Aspartate aminotransferase, U/L	38.0 (26.0‐59.0)	31.5 (20.0‐55.3)	38.0 (27.8‐59.0)	.075
Albumin, g/L	31.1 (27.9‐34.0)	33.2 (29.4‐36.8)	30.8 (27.9‐33.6)	.020
Total bilirubin, μmol/L	12.1 (8.4‐17.3)	10.6 (7.6‐14.2)	12.3 (8.9‐17.9)	.055
Lactate dehydrogenase, U/L	487.0 (375.5‐655.0)	433.5 (318.5‐547.5)	497.4 (384.3‐674.0)	.011
Blood urea nitrogen, mmol/L	7.8 (5.4‐11.5)	5.5 (3.9‐8.3)	8.2 (5.8‐11.8)	<.001
Creatinine, μmol/L	85.0 (66.0‐109.0)	72.5 (59.5‐86.8)	87.5 (67.0‐111.0)	.010
Uric acid, μmol/L	259.5 (183.0‐356.5)	223.0 (165.3‐356.5)	263.5 (184.8‐358.5)	.202
Prothrombin time, s	15.1 (14.0‐16.6)	14.3 (13.5‐15.5)	15.2 (14.3‐16.7)	.003
Activated partial thromboplastin time, second	39.3 (35.3‐45.4)	36.9 (34.5‐41.4)	39.6 (35.9‐45.5)	.051
D‐dimer, μg/mL	4.32 (1.39‐21.00)	2.30 (0.96‐7.10)	4.74 (1.60‐21.00)	.012
High‐sensitivity cardiac troponin I, pg/Ml	29.6 (10.2‐194.7)	8.5 (5.1‐19.0)	38.9 (11.5‐226.6)	<.001
NT‐proBNP, pg/mL	826.0 (313.3‐2128.0)	503.0 (160.0‐1121.5)	874.0 (384.0‐2418.0)	.006
C reactive protein, mg/L	100.0 (61.6‐156.5)	77.0 (51.8‐120.7)	104.3 (62.8‐174.2)	.010
Erythrocyte sedimentation rate, mm/hour	39.0 (20.5‐66.0)	47.5 (29.0‐72.0)	38.0 (20.0‐65.0)	.263
Ferritin, μg/L	1386.1 (795.4‐2077.4)	849.9 (489.9‐1472.2)	1478.1 (884.1‐2401.7)	.002
Procalcitonin, ng/mL	0.11 (0.21‐0.63)	0.10 (0.05‐0.19)	0.23 (0.12‐0.78)	<.001
Interleukin‐1β, pg/mL	5.0 (5.0‐5.0)	5.0 (5.0‐5.0)	5.0 (5.0‐5.0)	.810
Interleukin‐2R, U/ml	1128.0 (812.0‐1556.5)	913.0 (532.8‐1423.8)	1177.0 (848.0‐1586.0)	.014
Interleukin‐6, pg/mL	51.2 (19.4‐135.6)	12.8 (2.7‐33.1)	59.4 (28.4‐145.2)	<.001
Interleukin‐8, pg/mL	25.8 (14.7‐55.6)	13.4 (5.0‐23.4)	29.8 (16.7‐68.3)	<.001
Interleukin‐10, pg/mL	8.3 (5.0‐15.0)	5.0 (5.0‐5.5)	9.2 (5.2‐16.3)	<.001
Tumor necrosis factor‐α, pg/mL	11.0 (7.4‐15.5)	6.8 (4.0‐13.9)	11.3 (8.0‐15.6)	.004

Data are median (IQR), n (%), or n/N (%). *P* values were calculated by the Mann‐Whitney *U* test, χ^2^ test, or Fisher's exact test, as appropriate. Abbreviation: SOFA, sequential organ failure assessment.

We found no association between glucocorticoids and death, the incidence of complications, the incidence of more than one complication, or the use of invasive mechanical ventilation/extracorporeal membrane oxygenation (ECMO) in the multivariate logistic regression analysis (Table S3). In the multivariable Cox regression model, glucocorticoid therapy failed to affect the survival time of patients in the noncritical group (*P *= .558，Table S4) or critical group (*P *= .113, Table S4; log‐rank *P *= .15, Figure S2). Incredibly, glucocorticoid treatment prolonged the hospital length of stay of both noncritical patients (HR [hazard ratio]= 0.563, 95% CI [confidence interval]: 0.504‐0.628, *P *< .001, after adjusting for age) and critical patients (HR = 0.080, 95% CI: 0.024‐0.262, *P *< .001). Kaplan‐Meier curves with log‐rank tests drew consistent conclusions (log‐rank *P *< .0001 for noncritical patients; log‐rank *P *< .0001 for critical patients) (Figure 1A,B). Furthermore, delayed viral shedding time in noncritical patients (HR = 0.892, 95% CI: 0.798‐0.997, *P *= .043) was observed after adjusting for age and time from illness onset to admission (Table S4). However, the Kaplan‐Meier curve showed no correlation between glucocorticoids and viral shedding time in either noncritical (log‐rank *P *= .49, Figure 1C) or critical patients (log‐rank *P *= .57, Figure 1D).

**FIGURE 1:**
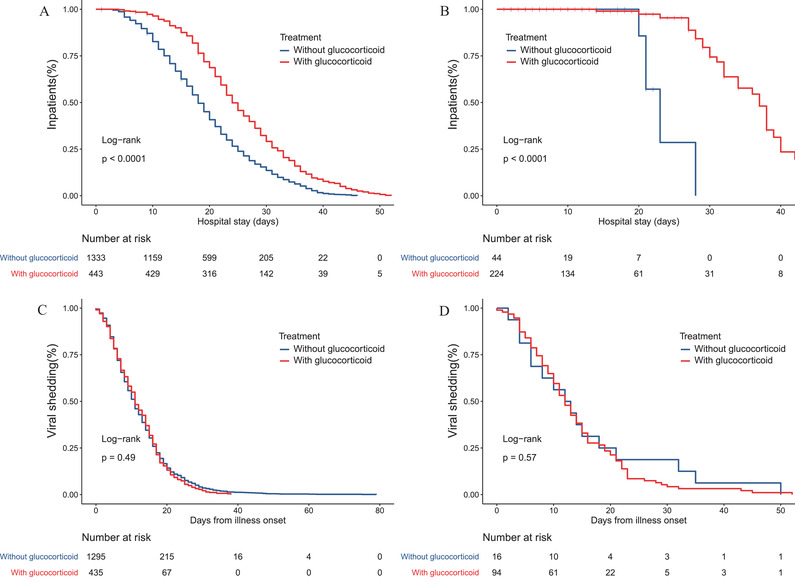
Kaplan‐Meier curves showing hospital length of stay and viral shedding time of patients with different disease severities. A, Using glucocorticoids prolonged the median hospital length of stay of patients in the noncritical group in log‐rank test (log‐rank *P *< .0001). B, Using glucocorticoids prolonged the median hospital length of stay of patients in the critical group in log‐rank test (log‐rank *P *< .0001). C, Using glucocorticoids did not prolong the median viral shedding time of patients in the noncritical group in log‐rank test (log‐rank *P *= .49). D, Using glucocorticoids did not prolong the median viral shedding time of patients in the critical group in log‐rank test (log‐rank *P *= .57)

Our research has several limitations. First, retrospective research has inherent limitations. However, compared with RCT, this study covered a wider population, including all confirmed patients. Second, all patients were located in Wuhan, China. Therefore, national or worldwide experience in treating COVID‐19 with glucocorticoids is needed to support our findings.

In conclusion, we conducted a real‐world study of the early administration of glucocorticoids in patients with COVID‐19 in Wuhan, China. Glucocorticoids were used in noncritically ill patients with unstable vital signs and the majority of critically ill patients. The use of glucocorticoids was related to prolonged hospitalization time of patients with different disease severities and prolonged viral shedding time of patients in the noncritical group. Glucocorticoids should be used with caution, especially in noncritical patients with older age and delayed admission. Physicians should prudently prescribe glucocorticoids according to the clinical guidelines and the actual situation of individual patients.

## ETHICS APPROVAL AND CONSENT TO PARTICIPATE

This study was approved by the Research Ethics Commission of Tongji Hospital of Huazhong University of Science and Technology (TJ‐IRB20200406) with written informed consent waived. The trial has been registered in Chinese Clinical Trial Registry (ChiCTR2000032161).

## AUTHOR CONTRIBUTIONS

Chunrui Li and Qinglei Gao had full access to all of the data in the study and take responsibility for the integrity of the data and the accuracy of the data analysis. Xiaofei Jiao, Ya Wang, Dan Liu, and Shaoqing Zeng equally contributed to this work. Dan Liu and Qinglei Gao designed the study. Jianhua Chi, Ruyuan Li, Yang Yu, Shaoqing Zeng, Ruidi Yu, Siyuan Wang, Yuan Yuan, Yue Gao, and Sen Xu acquired, analyzed, and interpreted the data. Xiaofei Jiao, Ya Wang, Dan Liu, and Shaoqing Zeng analyzed and interpreted data, and wrote the paper. Chunrui Li and Qinglei Gao provided critical revision of the manuscript for important intellectual content and administrative, technical, or material support. Chunrui Li and Qinglei Gao supervised this work. All authors vouch for the respective data and analysis, approved the final version, and agreed to publish the manuscript.

## CONFLICT OF INTEREST

The authors declare that there is no conflict of interest that could be perceived as prejudicing the impartiality of the research reported.

## Supporting information

Supporting Information

## Data Availability

Data supporting the findings of this study are available from the corresponding author upon reasonable request. The data containing information that could compromise research participant privacy, and so are not publicly available.

## References

[1] Kox M , Waalders NJB , Kooistra EJ , Gerretsen J , Pickkers P . Cytokine levels in critically Ill patients with COVID‐19 and other conditions. JAMA. 2020;324(15):1565‐1567. 10.1001/jama.2020.17052 PMC7489366 32880615

[2] Mehta P , McAuley DF , Brown M , Sanchez E , Tattersall RS , Manson JJ . COVID‐19: consider cytokine storm syndromes and immunosuppression. Lancet. 2020;395(10229):1033‐1034. 32192578 10.1016/S0140-6736(20)30628-0 PMC7270045

[3] Wiersinga WJ , Rhodes A , Cheng AC , Peacock SJ , Prescott HC . Pathophysiology, transmission, diagnosis, and treatment of coronavirus disease 2019 (COVID‐19): a review. JAMA. 2020;324(8):782‐793. 32648899 10.1001/jama.2020.12839

[4] Sanders JM , Monogue ML , Jodlowski TZ , Cutrell JB . Pharmacologic treatments for coronavirus disease 2019 (COVID‐19): a review. JAMA. 2020;323(18):1824‐1836. 32282022 10.1001/jama.2020.6019

[5] Zhou W , Liu Y , Tian D , et al. Potential benefits of precise corticosteroids therapy for severe 2019‐nCoV pneumonia. Signal Transduct Target Ther. 2020;5:18. 32296012 10.1038/s41392-020-0127-9 PMC7035340

[6] Wang Y , Jiang W , He Q , et al. A retrospective cohort study of methylprednisolone therapy in severe patients with COVID‐19 pneumonia. Signal Transduct Target Ther. 2020;5(1):57. 32341331 10.1038/s41392-020-0158-2 PMC7186116

[7] Hu Y , Wang T , Hu Z , et al. Clinical efficacy of glucocorticoid on the treatment of patients with COVID‐19 pneumonia: a single‐center experience. Biomed Pharmacother. 2020;130:110529. 32736237 10.1016/j.biopha.2020.110529 PMC7386262

[8] Huang R , Zhu C , Jian W , et al. Corticosteroid therapy is associated with the delay of SARS‐CoV‐2 clearance in COVID‐19 patients. Eur J Pharmacol. 2020;889:173556. 32941927 10.1016/j.ejphar.2020.173556 PMC7490250

[9] Zha L , Li S , Pan L , et al. Corticosteroid treatment of patients with coronavirus disease 2019 (COVID‐19). Med J Aust. 2020;212(9):416‐420. 32266987 10.5694/mja2.50577 PMC7262211

[10] Horby P , Lim WS , Emberson JR , et al. Dexamethasone in hospitalized patients with covid‐19 ‐ preliminary report. N Engl J Med. 2020 10.1056/NEJMoa2021436. PMC7383595 32678530

