# Supplementary material for: A real‐world study of glucocorticoid treatment in COVID‐19 patients with different disease severities
Source: Clin Transl Med. 2020 Dec 8;10(8):e235. doi: 10.1002/ctm2.235 (PMC7724098; doi:10.1002/ctm2.235)
Supplement: Supplementary file 1 — Supporting Information [file CTM2-10-e235-s001.docx]

**Supplemental Materials**

**Supplemental Methods**

**1. Study design and participants**

This is a retrospective, multicenter real-world study performed in the Sino-French New City campus and the Optical Valley Campus of Tongji hospital, two of the largest designated hospitals in Wuhan, to receive patients with COVID-19.

A total of 2,387 patients had been discharged or died in two campuses from January 27 to March 21, 2020. The follow-up ended on March 21. Among them, 72 patients did not meet the diagnosis of COVID-19, according to the 7th edition of the Diagnosis and Treatment Protocol of COVID-19 by the National Health Commission. We further excluded 205 patients that were transferred from mobile cabin hospitals for the requirement of isolation, 31 patients that died within 24 hours of admission, and 35 patients that were under 18 years old, pregnancy, re-hospitalization or discharged for special reasons such as dialysis. Finally, 2,044 patients were included in this study.

**2. Data collection**

All COVID-19 patients were classified into the noncritical group or critical group based on the most severe condition during the entire course of the disease, according to the Guidance for Coronavirus Disease 2019 (7th edition) released by the National Health Commission of China. Critical COVID-19 patients refer to those who meet any of the following criteria: 1) respiratory failure and need mechanical ventilation; 2) the occurrence of shock; 3) complicated with other organ failures, need to stay in the intensive care unit. Epidemiological, demographic, laboratory, radiological, treatment, and outcome data were collected from electronic medical records and recorded into a standardized predesigned database. A trained team of physicians extracted and analyzed the data. All information obtained from the electronic medical records was cross-checked by two physicians.

**3. Laboratory confirmation of SARS-CoV-2**

Tongji Hospital was officially approved by the National Health Commission for confirmation of diagnosis. The reverse-transcription polymerase chain reaction (RT-PCR) assay was carried out according to the protocol recommended by the World Health Organization. Commercialized RNA extraction kits (Biogerm, Shanghai, China) were used to extract total RNA. Two targets were used in real time RT-PCR. Target 1: open reading frame 1 ab (ORF1ab), forward primer: 5′-CCCTGTGGGTTTTACACTTAA-3′, reverse primer: 5′-ACGATTGTGCATCAGCTGA-3′, and probe: 5'-VIC-CCGTCTGCGGTATGTGGAAAGGTTATGG-BHQ1-3'. Target 2: For the N region, forward primer: 5′-GGGGAACTTCTCCTGCTAGAAT-3′, reverse primer: 5′-CAGACATTTTGCTCTCAAGCTG-3′, and the probe: 5’-FAM-TTGCTGCTGCTTGACAGATT-TAMRA-3’. The real time RT-PCR assay was carried out with a SARS-CoV-2 nucleic acid detection kit according to the manufacturer’s protocol (Shanghai Bio-germ Medical Technology company). The exact protocol was previous reported^1^.

**4. Diagnosed and disease severity of COVID-19**

Documented COVID-19 was defined based on epidemiological history, clinical manifestations (fever and/or respiratory symptoms; laboratory evidence of normal or decreased number of leukocytes and/or lymphopenia), lung imaging, and positive results of SARS-CoV-2 nucleic acid detection and/or detection of serum-specific antibodies added in 7^th^ edition^2^.

Clinically diagnosed patients^2^: The clinically diagnosed cases were only allowed for the cases in the Hubei Province for the period of February 9 to 19 based on the Diagnosis and Treatment guidance of COVID-19 (5^th^ edition) released by the National Health Commission of China on February 8, 2020 and abolished on February 19, 2020. A presumptive case was defined as meeting the following criteria: (1) recent travel history to Wuhan City or Hubei Province; or close contact with a confirmed or probable case; or cluster transmission; (2) fever and/or respiratory symptoms; (3) laboratory evidence of normal or decreased number of leukocytes and/or lymphopenia. Those presumptive cases with further radiographic evidence showing pneumonia but without a positive RT-PCR test result were defined as clinically diagnosed cases.

Discharge criteria: meeting criteria as fellows: temperature returned to normal for at least three days; respiratory symptoms improved significantly; radiology showed that acute exudative lesions were improved significantly; with two consecutive negative results of SARS-CoV-2 in sputum or nasopharynx swabs or other respiratory tract specimens (interval of at least 24 hours).

**5. Outcomes and definitions**

The primary outcome was death. Secondary outcomes included hospital length of stay, time from illness onset to discharge or death, viral shedding time, the incidence of complications, and receiving invasive mechanical ventilation/ECMO.

Fever was defined as axillary temperature of at least 37.3°C. The SOFA (Sepsis–related Organ Failure Assessment) score was used to describe organ dysfunction/failure^3^. The illness severity of COVID–19 was defined according to the 7th edition of the Diagnosis and Treatment Protocol of COVID–19. Sepsis and septic shock were defined according to the 2016 Third International Consensus Definition for Sepsis and Septic Shock^4^. Secondary infection was diagnosed when patient had a positive culture of a new pathogen was obtained from lower respiratory tract specimens (qualified sputum, endotracheal aspirate, or bronchoalveolar lavage fluid) or blood samples after admission. Acute kidney injury was diagnosed according to the KDIGO clinical practice guidelines and acute respiratory distress syndrome (ARDS) was diagnosed according to the Berlin Definition^5^. Acute cardiac injury was diagnosed if serum level of cardiac troponin I was above the 99th percentile upper reference limit. Coagulopathy was defined as a 3–second extension of prothrombin time or a 10–second extension of activated partial thromboplastin time. Hypoproteinemia was defined as blood albumin of less than 30 g/L. Heart failure was diagnosed based on the established Framingham criteria^6^. Acute liver injury was defined as serum level of total bilirubin ≥ 3 mg/dl and an acute increase in ALT of at least five times the upper limit of the normal range and/or an increase in alkaline phosphatase of at least twice the upper limit of the normal^1^.The criteria for discharge require absence of fever for at least 3 days, substantial improvement in both lungs in CTs, clinical remission of respiratory symptoms and two throat–swab samples negative for SARS–CoV–2 RNA detection obtained at least 24h apart^1^.

**6. Statistical analysis**

Continuous and categorical variables were presented as median (IQR) and n (%), respectively. Mann-Whitney U test, χ² test, or Fisher’s exact test were used to compare differences between groups where appropriate. The multivariable logistic regression model was used to explore the effect of glucocorticoids on death, complications, and requiring invasive mechanical ventilation/ECMO. The odds ratio (OR) along with the 95% confidence interval (95%CI) were reported. Kaplan-Meier curve and Cox regression analysis with death, discharge, and viral shedding as endpoints were employed between patients with different use of glucocorticoids. A two-sided α of less than 0.05 was considered statistically significant. Statistical analyses were done using the SPSS software (version 23.0).

**References**

1. Chen T, Wu D, Chen H, Yan W, Yang D, Chen G, et al. Clinical characteristics of 113 deceased patients with coronavirus disease 2019: retrospective study. *Bmj.* 2020;368:m1091.

2. A P, L L, C W, H G, X H, Q W, et al. Association of Public Health Interventions With the Epidemiology of the COVID-19 Outbreak in Wuhan, China. 2020.

3. Vincent JL, Moreno R, Takala J, Willatts S, De Mendonça A, Bruining H, et al. The SOFA (Sepsis-related Organ Failure Assessment) score to describe organ dysfunction/failure. On behalf of the Working Group on Sepsis-Related Problems of the European Society of Intensive Care Medicine. *Intensive Care Med.* 1996;22(7):707-10.

4. Huang C, Wang Y, Li X, Ren L, Zhao J, Hu Y, et al. Clinical features of patients infected with 2019 novel coronavirus in Wuhan, China. *Lancet.* 2020;395(10223):497-506.

5. Ranieri VM, Rubenfeld GD, Thompson BT, Ferguson ND, Caldwell E, Fan E, et al. Acute respiratory distress syndrome: the Berlin Definition. *JAMA.* 2012;307(23):2526-33.

6. McKee PA, Castelli WP, McNamara PM, Kannel WB. The natural history of congestive heart failure: the Framingham study. *N Engl J Med.* 1971;285(26):1441-6.

**Supplemental data**

| **Table S1. Baseline characteristics of patients in noncritical group and critical group.** | | | | |
| --- | --- | --- | --- | --- |
|  | Total (N=2044) | Noncritical (N=1776) | Critical (N=268) | *P* value |
| **Demographic and clinical characteristics** | | | | |
| Age, years | 62.0 (51.0-70.0) | 61.0 (49.0-69.0) | 69.0 (62.0-77.0) | <0.001 |
| ≥60 | 1177 (57.58%) | 984 (54.4%) | 193 (82.1%) | <0.001 |
| Sex |  |  |  | <0.001 |
| Female | 1044 (51.08%) | 952 (53.6%) | 92 (34.3%) |  |
| Male | 1000 (48.92%) | 824 (46.4%) | 176 (65.7%) |  |
| Presence of comorbidity | 1175/2039 (57.63%) | 963/1773 (54.3%) | 212/266 (79.7%) | <0.001 |
| Hypertension | 810/2039 (39.73%) | 661/1773 (37.3%) | 149/266 (56.0%) | <0.001 |
| Diabetes | 341/2039 (16.72%) | 281/1773 (15.8%) | 60/266 (22.6%) | 0.007 |
| Coronary heart disease | 199/2039 (9.76%) | 153/1773 (8.6%) | 46/266 (17.3%) | <0.001 |
| Number of comorbidities | 1 (0-2) | 1 (0-1) | 1 (1-2) | <0.001 |
| Temperature, °C | 38.3 (37.5-39.0) | 38.2 (37.5-39.0) | 38.3 (37.8-38.9) | 0.211 |
| Fever | 1644/2041 (80.55%) | 1423/1774 (80.2%) | 221/267 (82.8%) | 0.362 |
| Cough | 1487/2041 (72.86%) | 1272/1774 (71.7%) | 215/267 (80.5%) | 0.003 |
| Dyspnea | 870/2041 (42.63%) | 689/1774 (38.8%) | 181/267 (67.8%) | <0.001 |
| Respiratory rate, per min | 21 (20-24) | 20 (20-22) | 28.0 (22-33) | <0.001 |
| Mean arterial pressure, mmHg | 96.67 (88.67-105.67) | 96.7 (88.7-105.3) | 97.0 (89.7-106.7) | 0.276 |
| SpO2, % | 95.0 (92.0-97.0) | 96.0 (93.0-97.0) | 86.0 (76.0-92.0) | <0.001 |
| SOFA score at admission | 1.0 (0.0-2.0) | 1.0 (0.0-1.0) | 5.0 (3.5-6.5) | <0.001 |
| 0-1 | 594/1211 (49.05%) | 1426 (80.3%) | 1 (0.4%) | <0.001 |
| 2-3 | 372/1211 (30.72%) | 306 (17.2%) | 66 (24.6%) |  |
| ≥4 | 245/1211 (20.23%) | 44 (2.5%) | 201 (75.0%) |  |
| Time from illness onset to hospital admission, days | 12.0 (8.0-18.0) | 12.0 (8.0-19.0) | 11.0 (7.0-16.0) | <0.001 |
| **Laboratory findings** | | | | |
| White blood cell count, × 10^9^ per L | 5.69 (4.41-7.38) | 5.42 (4.32-6.88) | 9.14 (6.29-12.92) | <0.001 |
| Lymphocyte count, × 10^9^ per L | 1.12 (0.77-1.56) | 1.22 (0.86-1.63) | 0.61 (0.42-0.83) | <0.001 |
| Hemoglobin, g/L | 126.0 (116.0-137.0) | 126.0 (116.0-137.0) | 128.0 (115.0-143.0) | 0.144 |
| Platelet count, × 10^9^ per L | 222.0 (167.0-291.5) | 227.0 (175.0-296.0) | 165.5 (116.7-236.0) | <0.001 |
| Alanine aminotransferase, U/L | 22.0 (14.5-37.0) | 22.0 (14.0-36.0) | 27.5 (18.0-43.0) | <0.001 |
| Aspartate aminotransferase, U/L | 25.0 (19.0-38.0) | 24.0 (18.0-34.0) | 39.0 (27.0-59.0) | <0.001 |
| Albumin, g/L | 35.5 (32.0-39.4) | 36.2 (32.8-39.9) | 31.0 (27.9-34.0) | <0.001 |
| Total bilirubin, μmol/L | 8.7 (6.5-12.1) | 8.4 (6.3-11.4) | 12.1 (8.5-17.5) | <0.001 |
| Lactate dehydrogenase, U/L | 262.0 (203.0-351.0) | 247.0 (197.0-313.0) | 480.5 (370.7-634.7) | <0.001 |
| Blood urea nitrogen, mmol/L | 4.4 (3.4-5.8) | 4.2 (3.3-5.3) | 7.9 (5.4-12.2) | <0.001 |
| Creatinine, μmol/L | 68.0 (57.0-85.0) | 67.0 (56.0-82.0) | 83.0 (66.0-109.0) | <0.001 |
| Uric acid, μmol/L | 255.3 (196.2-332.0) | 255.0 (198.0-317.4) | 263.0 (184.3-363.0) | 0.240 |
| Prothrombin time, s | 13.8 (13.3-14.5) | 13.7 (13.2-14.3) | 15.1 (14.1-16.6) | <0.001 |
| Activated partial thromboplastin time, s | 38.8 (35.8-42.7) | 38.7 (35.8-42.3) | 39.7 (35.8-45.4) | 0.005 |
| D-dimer, μg/mL | 0.73 (0.35-1.74) | 0.61 (0.31-1.33) | 4.16 (1.39-21.00) | <0.001 |
| High-sensitivity cardiac troponin I, pg/mL | 4.4 (1.9-10.6) | 3.6 (1.9-7.6) | 29.1 (9.1-183.1) | <0.001 |
| NT-proBNP, pg/mL | 119.0 (44.5-353.0) | 93.5 (36.0-231.3) | 768.0 (290.3-2194.8) | <0.001 |
| C reactive protein, mg/L | 17.2 (2.5-64.8) | 10.9 (2.0-48.4) | 98.2 (59.2-152.0) | <0.001 |
| Erythrocyte sedimentation rate, mm/h | 32.0 (15.0-58.5) | 30.0 (14.0-57.0) | 40.0 (21.5-67.0) | <0.001 |
| Ferritin, ug/L | 554.7 (313.8-1067.3) | 488.7 (284.4-824.6) | 1353.2 (798.0-2162.4) | <0.001 |
| Procalcitonin, ng/mL | 0.06 (0.04-0.11) | 0.05 (0.03-0.08) | 0.21 (0.11-0.68) | <0.001 |
| Interleukin-1β, pg/mL | 5.0 (5.0-5.0) | 5.0 (5.0-5.0) | 5.0 (5.0-5.0) | 0.997 |
| Interleukin-2R, U/ml | 570.0 (363.0-871.0) | 526.0 (342.0-759.0) | 1098.0 (768.0-1565.0) | <0.001 |
| Interleukin-6, pg/mL | 5.37 (2.02-22.45) | 4.27 (1.77-14.75) | 54.88 (22.00-137.10) | <0.001 |
| Interleukin-8, pg/mL | 10.7 (6.1-20.9) | 9.7 (5.7-17.8) | 25.7 (13.8-55.5) | <0.001 |
| Interleukin-10, pg/mL | 5.0 (5.0-5.7) | 5.0 (5.0-5.0) | 8.6 (5.0-14.9) | <0.001 |
| Tumor necrosis factor-α, pg/mL | 7.7 (5.8-10.3) | 7.5 (5.6-9.7) | 11.1 (7.4-15.5) | <0.001 |

Data are median (IQR), n (%) or n/N (%). p values were calculated by Mann-Whitney U test, χ² test or Fisher’s exact test, as appropriate. SOFA, Sequential Organ Failure Assessment.

| **Table S2. Treatments and outcomes of patients with different severities.** | | | | |
| --- | --- | --- | --- | --- |
|  | Total (N=2044) | Noncritical (N=1776) | Critical (N=268) | *P* value |
| **Treatments** | | | | |
| Glucocorticoids | 667 (32.6%) | 443 (24.9%) | 224 (83.6%) | <0.001 |
| Time from illness onset to using glucocorticoids, days | 2.0 (1.0-4.0) | 2.0 (1.0-4.0) | 1.0 (1.0-3.0) | 0.002 |
| Duration of using glucocorticoids, days | 7.0 (4.0-12.0) | 8.0 (5.0-12.0) | 5.0 (3.0-10.0) | <0.001 |
| Antibiotics | 1618/2043 (79.2%) | 1359 (76.5%) | 259/267 (97.0%) | <0.001 |
| Antiviral treatments | 1892 (92.6%) | 1672 (94.1%) | 220 (82.1%) | <0.001 |
| Intravenous immunoglobin | 468 (22.9%) | 320 (18.0%) | 148 (55.2%) | <0.001 |
| High-flow nasal cannula oxygen therapy | 75/2039 (3.7%) | 64/1771 (3.6%) | 11 (4.1%) | 0.727 |
| Non-invasive mechanical ventilation | 117/2039 (5.7%) | 0 (0.00%) | 117 (43.7%) | <0.001 |
| Invasive mechanical ventilation | 122/2039 (6.0%) | 1/1771 (0.1%) | 121 (45.1%) | <0.001 |
| Duration of mechanical ventilation, days | 5.0 (2.0-9.0) | 9.0 (9.0-9.0) | 5.0 (2.0-9.0) | 0.485 |
| ECMO | 7/2039 (0.3%) | 0 (0.00%) | 7 (2.6%) | <0.001 |
| Duration of ECMO, days | 9.0 (1.0-18.0) | - † | 9.0 (1.0-18.0) | - † |
| **Outcomes** | | | | |
| The highest SOFA Score | 1.0 (0.0-2.0) | 1.0 (0.0-2.0) | 14.0 (12.0-17.0) | <0.001 |
| 0-1 | 1321 (64.6%) | 1321 (73.0%) | 0 (0.0%) | <0.001 |
| 2-3 | 385 (18.8%) | 384 (21.2%) | 1 (0.4%) | ‥ |
| ≥4 | 338 (16.5%) | 104 (5.7%) | 234 (99.6%) | ‥ |
| Glucose | 6.1 (5.2-7.7) | 5.9 (5.2-7.3) | 7.7 (6.3-10.9) | <0.001 |
| Acute liver injury | 773 (37.8%) | 631 (35.5%) | 142 (53.0%) | <0.001 |
| Sepsis | 711 (34.8%) | 443 (24.9%) | 268 (100%) | <0.001 |
| Hypoproteinemia | 474/2041 (23.3%) | 276/1773 (15.6%) | 198 (73.9%) | <0.001 |
| ARDS | 446 (21.8%) | 178 (10.0%) | 268 (100.0%) | <0.001 |
| Acute cardiac injury | 309/1831 (16.9%) | 117/1575 (7.4%) | 192/256 (75.0%) | <0.01 |
| Respiratory failure | 273 (13.4%) | 18 (1.0%) | 255 (95.1%) | <0.001 |
| Acute kidney injury | 250 (12.2%) | 122 (6.9%) | 128 (47.8%) | <0.001 |
| Coagulopathy | 243 (11.9%) | 87 (4.9%) | 156 (58.2%) | <0.001 |
| Septic shock | 242 (11.8%) | 12 (0.7%) | 230 (85.8%) | <0.001 |
| Admission to ICU | 163 (8.0%) | 8 (0.5%) | 15 (57.8%) | <0.001 |
| Heart failure | 134/2041 (6.6%) | 13/1774 (0.7%) | 121/267 (45.3%) | <0.001 |
| Secondary infection | 27 (1.3%) | 7 (0.4%) | 20 (7.5%) | <0.001 |
| Time from illness onset to ICU admission, days | 15.0 (11.0-22.0) | 14.0 (12.0-27.0) | 15.0 (11.0-22.0) | 0.727 |
| ICU length of stay, days | 7.0 (3.0-11.0) | 10.5 (2.5-17.5) | 7.0 (3.0-11.0) | 0.330 |
| Time from illness onset to discharge, days | 35.0 (28.0-43.0) | 35.0 (28.0-43.0) | 46.0 (38.0-51.0) | <0.001 |
| Time from illness onset to death, days | 21.0 (16.0-30.0) | 22.0 (10.5-54.5) | 21.0 (16.0-30.0) | 0.796 |
| Hospital length of stay of survivors, days | 20.0 (14.0-28.0) | 20.0 (14.0-27.0) | 32.0 (27.0-38.0) | <0.001 |
| Hospital length of stay of nonsurvivors, days | 9.0 (5.0-16.0) | 9.0 (5.5-15.8) | 9.0 (5.0-16.0) | 0.860 |
| Duration of viral shedding after COVID-19 onset, days | 11.0 (6.0-17.0) | 11.0 (6.0-17.0) | 7.0 (12.0-19.0) | 0.103 |
| Nonsurvivors | 235 (11.5%) | 6 (0.3%) | 229 (85.4%) | <0.001 |

Data are median (IQR), n (%) or n/N (%). p values were calculated by the Mann-Whitney U test, χ² test, or Fisher’s exact test, as appropriate. ECMO, extracorporeal membrane oxygenation; SOFA, Sequential Organ Failure Assessment; ARDS, acute respiratory distress syndrome; ICU, intensive care unit; COVID-19, coronavirus disease 2019.

† The Mann-Whitney U test cannot be conducted because of no patients in the noncritical group received ECMO.

| **Table S3. Risk factors of death, incidence of complications, incidence of more than one complication, and requirement of invasive mechanical ventilation/ECMO in two groups.** | | | |
| --- | --- | --- | --- |
|  | OR | 95%CI | *P* value |
| Death |  |  |  |
| Critical group |  |  |  |
| White blood cell count | 1.274 | 1.027-1.581 | 0.028 |
| Platelet count | 0.993 | 0.986-0.999 | 0.020 |
| C reactive protein | 1.014 | 1.004-1.025 | 0.009 |
| Interleukin-10 | 1.283 | 1.053-1.565 | 0.014 |
| Incidence of complications |  |  |  |
| Noncritical group |  |  |  |
| SOFA score at admission | 5.980 | 3.636-9.836 | <0.001 |
| Alanine aminotransferase | 1.095 | 1.065-1.127 | <0.001 |
| Activated partial thromboplastin time | 1.056 | 1.000-1.115 | 0.049 |
| High-sensitivity cardiac troponin I | 1.080 | 1.013-1.150 | 0.018 |
| Interleukin-2R | 1.001 | 1.000-1.002 | 0.025 |
| Incidence of more than one complication |  |  |  |
| Noncritical group |  |  |  |
| SOFA score at admission | 4.667 | 3.105-7.013 | <0.001 |
| Alanine aminotransferase | 1.014 | 1.004-1.024 | 0.005 |
| Albumin | 0.793 | 0.731-0.860 | <0.001 |
| Blood urea nitrogen | 1.317 | 1.097-1.580 | 0.003 |
| Activated partial thromboplastin time | 1.072 | 1.021-1.124 | 0.005 |
| Erythrocyte sedimentation rate | 0.986 | 0.974-0.997 | 0.017 |
| Requirement of invasive mechanical ventilation/ECMO |  |  |  |
| Critical group |  |  |  |
| Lymphocyte count | 0.312 | 0.102-0.958 | 0.042 |
| Prothrombin time | 1.278 | 1.026-1.591 | 0.028 |

OR, odds ratio. CI, confidence interval. SOFA, Sequential Organ Failure Assessment. ECMO, extracorporeal membrane oxygenation.

| **Table S4. Risk factors of prolonged hospital length of stay, viral shedding time and survival time.** | | | |
| --- | --- | --- | --- |
|  | HR | 95%CI | *P* value |
| Hospital length of stay |  |  |  |
| Noncritical group |  |  |  |
| Glucocorticoid treatment | 0.563 | 0.504-0.628 | <0.001 |
| Age | 0.986 | 0.983-0.990 | <0.001 |
| Critical group |  |  |  |
| Glucocorticoid treatment | 0.080 | 0.024-0.262 | <0.001 |
| Viral shedding time |  |  |  |
| Noncritical group |  |  |  |
| Glucocorticoid treatment | 0.892 | 0.798-0.997 | 0.043 |
| Age | 0.993 | 0.990-0.996 | <0.001 |
| Time from illness onset to admission | 0.965 | 0.959-0.970 | <0.001 |
| Survival time |  |  |  |
| Noncritical group |  |  |  |
| Glucocorticoid treatment | - | - | 0.558 |
| Critical group |  |  |  |
| Glucocorticoid treatment | - | - | 0.113 |

HR, hazard ratio; CI, confidence interval.

**Figure S1. The flowchart of this study.**





**Figure S2. Kaplan-Meier curve showing survival time of patients in the critical group with different use of glucocorticoids.**


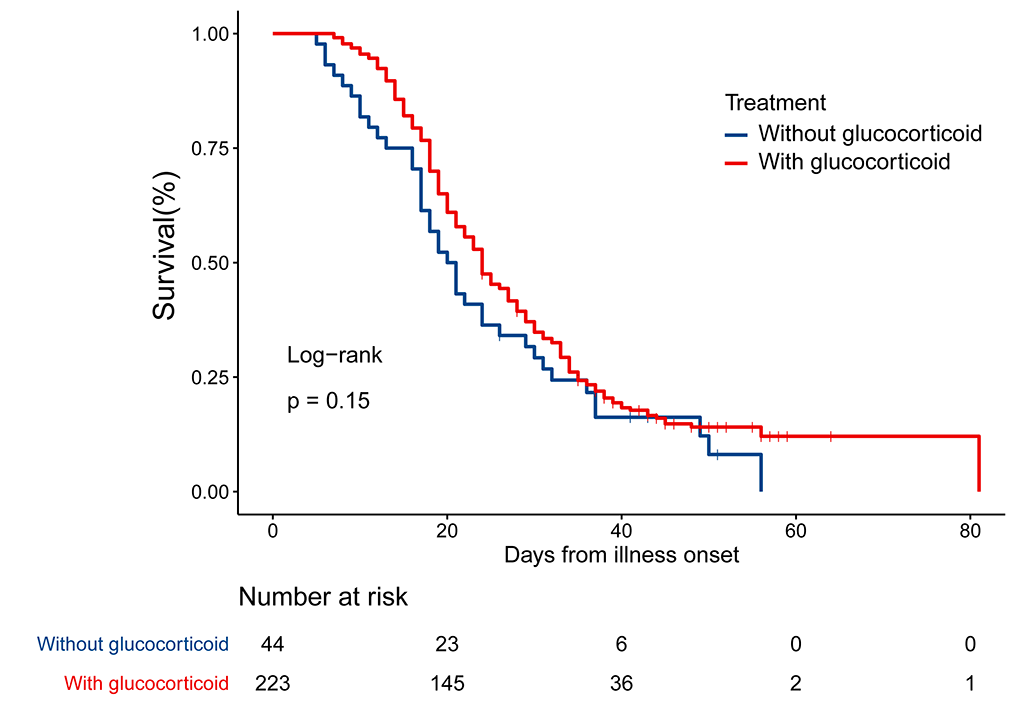


Using glucocorticoids did not prolong the median survival time of patients in the critical group in log-rank test (log-rank *P* = 0.15).
